# Allosteric Coupling Between Drug Binding and the Aromatic Cassette in the Pore Domain of the hERG1 Channel: Implications for a State-Dependent Blockade

**DOI:** 10.3389/fphar.2020.00914

**Published:** 2020-06-30

**Authors:** Meruyert Kudaibergenova, Jiqing Guo, Hanif M. Khan, Farhan Zahid, James Lees-Miller, Sergei Yu. Noskov, Henry J. Duff

**Affiliations:** ^1^ Centre for Molecular Simulation, Department of Biological Sciences, University of Calgary, Calgary, AB, Canada; ^2^ Cumming School of Medicine, Libin Cardiovascular Institute of Alberta, University of Calgary, Calgary, AB, Canada

**Keywords:** hERG1, dofetilide, ivabradine, docking, drug block

## Abstract

Human-ether-a-go-go-related channel (hERG1) is the pore-forming domain of the delayed rectifier K^+^ channel in the heart which underlies the *I_Kr_* current. The channel has been extensively studied due to its propensity to bind chemically diverse group of drugs. The subsequent hERG1 block can lead to a prolongation of the QT interval potentially leading to an abnormal cardiac electrical activity. The recently solved cryo-EM structure featured a striking non-swapped topology of the Voltage-Sensor Domain (VSD) which is packed against the pore-domain as well as a small and hydrophobic intra-cavity space. The small size and hydrophobicity of the cavity was unexpected and challenges the already-established hypothesis of drugs binding to the wide cavity. Recently, we showed that an amphipathic drug, ivabradine, may favorably bind the channel from the lipid-facing surface and we discovered a mutant (M651T) on the lipid facing domain between the VSD and the PD which inhibited the blocking capacity of the drug. Using multi-microseconds Molecular Dynamics (MD) simulations of wild-type and M651T mutant hERG1, we suggested the block of the channel through the lipid mediated pathway, the opening of which is facilitated by the flexible phenylalanine ring (F656). In this study, we characterize the dynamic interaction of the methionine-aromatic cassette in the S5-S6 helices by combining data from electrophysiological experiments with MD simulations and molecular docking to elucidate the complex allosteric coupling between drug binding to lipid-facing and intra-cavity sites and aromatic cassette dynamics. We investigated two well-established hERG1 blockers (ivabradine and dofetilide) for M651 sensitivity through electrophysiology and mutagenesis techniques. Our electrophysiology data reveal insensitivity of dofetilide to the mutations at site M651 on the lipid facing side of the channel, mirroring our results obtained from docking experiments. Moreover, we show that the dofetilide-induced block of hERG1 occurs through the intracellular space, whereas little to no block of ivabradine is observed during the intracellular application of the drug. The dynamic conformational rearrangement of the F656 appears to regulate the translocation of ivabradine into the central cavity. M651T mutation appears to disrupt this entry pathway by altering the molecular conformation of F656.

## Introduction

The human-*ether-a-go-go*-related gene (hERG1) encodes a homo-tetrameric potassium channel found in excitable cells such as neurons in the brain (Wymore et al., 1997; Papa et al., 2003), adrenal glands, heart and smooth muscle tissues throughout the human body (Warmke and Ganetzky, 1994; Wymore et al., 1997; Farrelly et al., 2003). The function of the channel is most studied in the cardiovascular system, where hERG1 encodes for the α-subunit of the rapid delayed rectifier K^+^ current (*I_kr_*), contributing to phase 3 of the cardiac action potential and the QT interval observed in electrocardiograms (Trudeau et al., 1995). Most common congenital mutations in hERG1 result in reduced *I_kr_* current, elongating the QT wave and potentially causing a Long QT Syndrome (Sanguinetti et al., 1995). Long QT syndrome is a serious condition related to *torsade de pointes* arrhythmias which may deteriorate into ventricular fibrillation and ultimately sudden cardiac death (Romano, 1965; Sanguinetti and Tristani-Firouzi, 2006). The promiscuous susceptibility of hERG1 block by structurally diverse drugs underpins an acquired Long QT syndrome, which was the cause of withdrawal of several medications from the market (Zimmermann et al., 1992). It was estimated that hundreds of patients suffered from sudden cardiac death and ventricular fibrillation due to the off-target block of hERG1, making the channel an object of intense pharmacological interest (Rampe et al., 1997). Mandatory hERG1 pre-screening protocols have been implemented in the early stages of drug development in attempt to prevent the dangerous cardiovascular side effects (Food and Drug Administration, 2005; Gintant et al., 2006). Elucidating the promiscuous block of the channel by drugs represents a major research question which may have a profound impact on human health and drug development.

Drug-induced susceptibility of the hERG1 block has historically been attributed to the presence of a high affinity binding site in the channel: *the wide water-filled intra-cellular cavity* (Drici and Barhanin, 2000; Mitcheson et al., 2000a; Mitcheson et al., 2000b; Sanguinetti and Tristani-Firouzi, 2006). For decades, it was believed that the unique, large size of the central cavity of the hERG1 allowed for the trapping of chemically diverse molecules after the closure of the activation gate (Drici and Barhanin, 2000). This large cavity was attributed to the lack of a *Pro-Val-Pro* motif, commonly found and conserved in other potassium family channels (Kv1–4). Instead, the motif in hERG1 is replaced with *Ile-Phe-Gly* (Vandenberg et al., 2012). The lack of prolines in the helix was believed to cause a wider central cavity in hERG1, contributing to drug trapping in the cavity (Fernandez et al., 2004). A plethora of site-directed mutagenesis and electrophysiology studies have demonstrated the importance of residues in the central cavity such as T623 and S624, which affect the high affinity binding of drugs (Helliwell et al., 2018). However, two aromatic residues in the central cavity have emerged as the most essential participants in the drug induced block of hERG1: Y652 and F656 (Lees-Miller et al., 2000; Mitcheson et al., 2000a; Perry et al., 2004). Mutagenesis studies have further shown that the aromatic rings and the hydrophobic surface area of F656 and not the hydroxyl groups of Y652 were a determining factor in the drug induced block of the channel (Sanguinetti et al., 2005). Both Y652 and F656 are part of the K^+^ conduction pathway below the selectivity filter. These aromatic residues were shown to contribute to high-affinity drug binding in a variety of structural models developed over the years using mammalian or bacterial K^+^ channels with known crystal structures as templates for modeling (Mitcheson et al., 2000a; Osterberg and Aqvist, 2005; Stary et al., 2010). Many drugs with a positively charged amino group were proposed to bind in the cavity presumably stabilized with cation-π or π- π interactions with the aromatic rings of Y652 and F656 (Vandenberg et al., 2001; Mitcheson, 2003). However, drug sensitivity to the mutations in positions 652 and 656 varied greatly. For instance: mexiletine (Gualdani et al., 2015), flecainide (Melgari et al., 2015b), vesnarinone (Kamiya et al., 2001), propafenone (Witchel et al., 2004), bepridil (Kamiya et al., 2006), thioridazine (Milnes et al., 2006), show more acute sensitivity to mutations at site F656 than at replacements made at the site Y652.

In 2017, the cryo-EM structure of hERG1 (PDB id: 5VA2) was resolved at 3.8 Å resolution revealing completely new and different domain arrangements: hERG1 features a striking non-swapped topology of the Voltage-Sensor Domain (VSD) packed against the Pore-Domain (PD) (Wang and Mackinnon, 2017). While previous homology models of Shakers/Kv1.4 chimeras predicted swapped topology between the VSD and PD due to the presence of a long alpha-helical S4-S5 linker (~10 amino acids), the new structure shows a short, few amino acids long, S4–S5 linker (Wang and Mackinnon, 2017). Surprisingly, the cryo-EM structure also revealed a constricted hydrophobic intra-cavity space in striking contrast to the large cavity proposed in the past, hence challenging the already-established hypothesis discussed earlier (Wang and Mackinnon, 2017). Moreover, studies on hERG1 blockers such as quinidine, dofetilide, and terfenadine have unexpectedly shown that cation-π interactions in the hERG1 induced block are not critical determinants in channel block implying that π stacking of the aromatic residues with the drugs is the major contributing factor (Macdonald et al., 2018). The additional stabilization of bound drugs may be aided by the aromatic side-chain of F557 located in the S5 helix (Saxena et al., 2016). The potential importance of the cation-π interactions due to the positive charge in blockers are yet to be considered. Shagufta et al. used targeted chemical modifications of dofetilide to create a library of analogs with increased the basicity of the tertiary amine, which leads in increased block potency (Shagufta et al., 2009). Wang et al. reported an enhanced hERG blockade at acidic pH values, which can also be attributed to the protonated (cationic) state of the blocker (Wang et al., 2016).

In 2015, a newly-released heart-rate reducing drug-ivabradine was shown to display off-target effects by blocking hERG1 at physiologically relevant concentrations and prolonging phase 3 repolarization in the heart (Melgari et al., 2015a; Lees-Miller et al., 2015). Lees-Miller et al. suggested that in addition to the already established high-affinity intra-cavity site, hERG1 might potentially have a lipid-facing binding pocket located near the position of M651 (Lees-Miller et al., 2015). The follow-up study investigated WT-hERG1 and M651X-hERG1 mutants to test the potential role of the protein-lipid interface between the VSD and the PD in the selective binding of ivabradine. Interestingly, the well-known hERG1 blocker dofetilide was insensitive to the M651T mutation (Perissinotti et al., 2019). It was proposed that ivabradine was accessing and blocking the intra-cellular cavity of the channel through a lipid mediated pathway whereas dofetilide was directly targeting and interacting with the intra-cellular cavity (Kudaibergenova et al., 2019; Perissinotti et al., 2019). There are striking differences between the measured water-hexane partitioning coefficients of ivabradine and dofetilide, providing additional support of the lipid-mediated pathway for ivabradine. The partition free energy for ivabradine favors its solubilization in a hydrophobic environment, whereas dofetilide partitions more favorably into the aqueous phase (Perissinotti et al., 2019). Other hydrophobic molecules such as ceramide, which is a secondary messenger of the sphingolipid family, and ω-3 and ω-6 polyunsaturated fatty acids tend to block hERG1, revealing potential importance of the lipid-mediated block of the channel (Guizy et al., 2005; Ganapathi et al., 2010). Machine-learning based analysis performed on hundreds of hERG1 blockers have further corroborated the importance of the lipophilicity of drugs in determining the hERG1-targeting properties (Wacker and Noskov, 2018).

In this work, we explore in further detail the dynamics of the hydrophobic cassette (F656, F557, Y652, and M651) in WT-hERG1 and M651T-hERG1 channels. We propose that F656 acts as a flexible gate that allows a passage of drugs into the central cavity of the channel in a drug-specific manner. To illustrate the importance of the conformational plasticity of F656 in drug induced hERG1 blockade, we performed experimental studies of dofetilide and ivabradine binding to WT-hERG1 and a number of single and double mutations testing role of the residues comprising hydrophobic cassette in WT-hERG1 channel. We also compared the pharmacologic potency of dofetilide and ivabradine when applied to the intracellular domain *via* the patch-clamp pipette experiments. In addition, we performed molecular docking of these drugs to the WT-hERG1 and the M651T mutant to elucidate the specific binding modes for ivabradine and dofetilide. These combined structural and functional studies strongly endorse an allosteric mechanism that couple the drug blockade of hERG1 currents and the conformational dynamics of the hydrophobic cassette. The proposed coupling between conformational dynamics of aromatic cassette may lend a structural support to the well-known state-dependence of the drug blockade of hERG1 (Stork et al., 2007).

## Methods

### Molecular Dynamics Simulations

The development and relaxation of the explicit membrane-embedded systems (open-states for WT-hERG1 and M651T-hERG1 based on the atomic coordinates deposited to PDB:5VA2) were described in our previous work (Perissinotti et al., 2019). The structure was truncated before the PAS and after the CNBD domains and the missing residues were modeled using a 3-step protocol utilizing ROSETTA (Bender et al., 2016; Perissinotti et al., 2019). The Anton 2 software version 1.27.0 from D. E. Shaw Research was used for production runs (1.2–3.5 µs) using the purpose-built Anton 2 supercomputer (Shaw et al., 2014a; Shaw et al., 2014b). A 2.5 fs time step was used with nonbonded long-range interactions computed every 6 fs using the RESPA multiple-time-step algorithm (Tuckerman et al., 1992). The protein-membrane system was equilibrated for 250 ns and then subjected to 1.2 to 3.5 μs MD runs. The CHARMM36m (Huang et al., 2017) force-field were implemented for protein dynamics and the latest CHARMM36 lipid parameters were used to describe lipid membrane dynamics (Klauda et al., 2010; Klauda et al., 2012). The protein was embedded in the 1-palmitoyl-2-oleoyl-sn-glycero-3-phosphocholine (POPC) lipid bilayer using CHARMM-GUI (Jo et al., 2008). The POPC lipid bilayer was composed of 380 lipids in total: 188 in the upper leaflet and 192 in the lower leaflet. CHARMM-NBFIX Lennard-Jones parameters for K^+^ and Cl^-^ were used to simulate counterions dynamics with the standard TIP3P model used for water molecules (Jorgensen et al., 1983; Noskov and Roux, 2008). The production runs were executed at 313.15 K in a semi-isotropic (NPat) ensemble. The multi-integrator (multigrator) algorithm (Lippert et al., 2013) developed in-house by D. E. Shaw Research was used for temperature and semi-isotropic pressure coupling (Shaw et al., 2014a; Shaw et al., 2014b). The time step for the production runs was set to 2 fs and trajectories were saved every 240 ps. Non-bonded and long-range electrostatic interactions were evaluated every 2 and 6 fs, respectively. Long-range electrostatics was calculated using the k-Gaussian Ewald method implemented to enhance performance on the ANTON2 platform (Shan et al., 2005; Shaw et al., 2014a; Shaw et al., 2014b). The original MD trajectories should become available to the public through the Pittsburgh Supercomputing Center ANTON2 program for WT and mutant forms of the hERG1 channel under Project #PSCA17021P (the release form has been completed and submitted by the authors of the current study).

### Molecular Drug Docking

#### Structure Preparations

**Figure 1:**
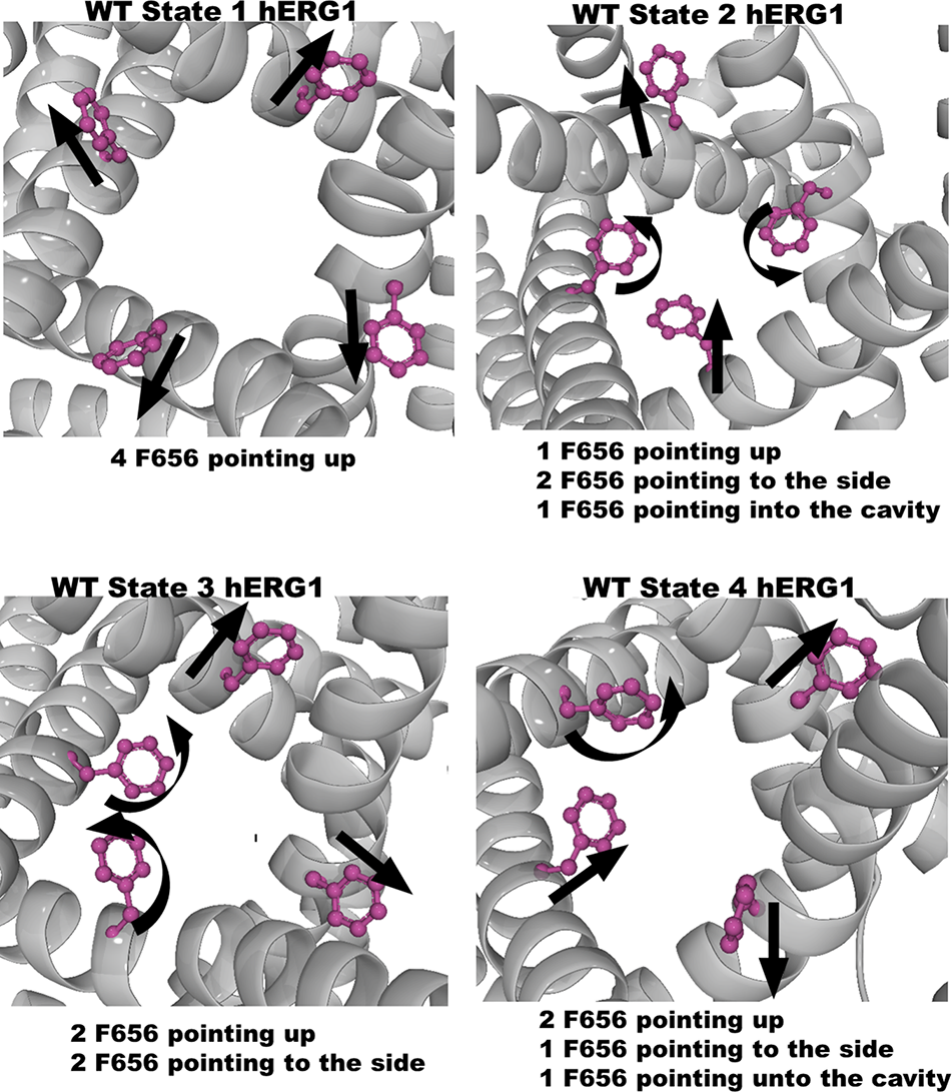
Four states of the WT-hERG1 extracted from the MD simulations based on the F656 geometric position in the central cavity. Top-down perspective of the central cavity is shown for each state. The channel is represented as a gray cartoon, F656 is shown as sticks and balls in purple. These states were used in the docking simulation of ivabradine and dofetilide to explore the differences in the binding affinity to the central cavity.

**Figure 2:**
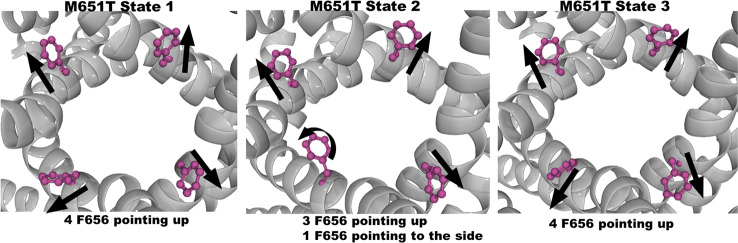
Three states of the M651T-hERG1 extracted from the MD simulations based on the F656 geometric position in the central cavity. Top-down perspective of the central cavity is shown for each state. The channel is represented as a gray cartoon, F656 is shown as sticks and balls in purple. These states were used in the docking simulation of ivabradine and dofetilide to explore the differences in the binding affinity to the central cavity.

The analysis of the equilibrium trajectories for WT-hERG1 and the M651T-hERG1 mutant identified several well-defined and well-populated conformational states of F656 (Perissinotti et al., 2019) in WT-hERG1 and M651T-hERG1 systems. This observation of F656 re-packing was corroborated in other studies (Negami et al., 2019; Yang et al., 2020). Distinct conformational states of F656 are represented by four WT-hERG1 and three M651T-hERG1 structures (Perissinotti et al., 2019) which were extracted from the production phase of the MD trajectories for molecular docking calculations. The WT-hERG1 system state 1 represents the relaxed cryo-EM equilibrated structure (MD production time: 0 μs). For the cryo-EM state relaxation, the harmonic constraints were applied to heavy atoms in protein backbone and side-chains as defined in the CHARMM-GUI membrane builder protocol with gradual release (steps 1 to 6 in CHARMM-GUI membrane builder) (Jo et al., 2008). The gradual relaxation in presence of harmonic constraints allows optimization of the side-chains positions and protein-lipid interactions while preserving overall fold of the protein captured in the cryo-EM state. After the relaxation, the structure features all four F656 in the same configuration (RMSD < 1.0 Å for Cα atoms) as observed in the original cryo-EM structure e.g. facing upwards (**Figure 1**). Accordingly, state 2 represents one of the preferred F656 orientations observed during multi micro-seconds MD simulation with two F656 facing the side of the cavity, one pointing upwards and one facing the central cavity (**Figure 1**). State 3 represents well-populated conformation with two F656 pointing upwards and two F656 facing the side of the cavity. Finally, structure used as state 4 contains one F656 pointing to the side of the cavity, one F656 facing the inside of the cavity, and two F656 pointing upwards (**Figure 1**). The MD simulations of M651T system features three distinct conformational states for F656 packing. State 1 of M651T system shows all four F656 in the same configuration, facing upwards (**Figure 2**). State 2 represents the structural ensemble with three F656 pointing upwards and one F656 facing side of the central cavity (**Figure 2**). State 3 is structural representative of the intra-cellular cavity with four F656 facing upwards (**Figure 2**). The receptor structures for each of the states were optimized using the Schrödinger's restrained minimization Protein Preparation Wizard (Sastry et al., 2013) and the positions of the hydrogen atoms were re-optimized.

Ligands for docking (dofetilide and ivabradine) were prepared using the Ligprep Wizard application available in the Schrödinger suites (Schrödinger, 2016). Ligprep restrained bond length, bond angles, converted 2D representation into 3D, added hydrogens, sampled ring conformations, minimized and optimized structures based on the OPLS force field (Jorgensen et al., 1996; Jorgensen and Tirado-Rives, 1988). Multiple ionization states of drugs and their relative abundance at a given pH have been proposed as an important factor for understanding the pH-dependent block of hERG1 (Wang et al., 2016). Charged states of ivabradine and dofetilide were generated using EPIK at a pH of 7 for drugs and using PROPKA at pH 7 for each of the conformational states considered for WT-hERG1 and M651T-hERG1. Neutral and charged states of both drugs yielded similar results within the uncertainties of the docking calculations used in this work and are shown in **Table S1** for both ionization states of the drug. Since the primary focus of this work was to investigate dynamics at the protein-lipid interface, we decided to focus on the neutral form of both compounds. The focus on the neutral state of two drugs is also in agreement with recent studies by Clancy and co-workers where the neutral states of two hERG blockers (dofetilide and moxifloxacin) were found to be more stable than the charged ones in the narrow intra-cavity space in the study employing exhaustive Umbrella-Sampling simulations (Yang et al., 2020). It is important to stress that computational and experimental studies showed that various charged-states of the blockers exist in a highly dynamical equilibrium, where preference for a specific charge state of the drug can be shifted by the environment (membrane, binding pocket, or aqueous phase) (Shagufta et al., 2009; Carvalho et al., 2013; Demarco et al., 2018; Perissinotti et al., 2019; Yue et al., 2019).

#### Docking Protocol

The Glide-XP (extra-precision) (Friesner et al., 2006) cross-docking modules of the Maestro suit in Schrödinger were used for all docking calculations with a ligand vdW scale factor of 0.80 and a RMSD cut-off of 2.0 Å. Ivabradine and dofetilide were docked to two locations in all receptors: the internal central cavity (defined by a geometric center between four F656 residues) and the lipid facing residues (between S6 helices) (**Figure S1**). For the internal cavity dockings, the Glide Grids for WT and M651T states were generated by selecting F656 and Y652, which have been implicated in the drug block of hERG1, of all four subunits as the centroid of the docking perimeter (**Figure S1**). As the channel is tetrameric, all four subunits were considered for each of the receptor states. The receptor grid dimensions are divided into inner and outer cubes. The length of the inner cube box edge was set to 10 Å and represents the space explored by Glide as acceptable positions for the geometrical center of the drugs. The outer box edge, representing the space all atoms of the drug must occupy, was set to 26 Å. The same receptor grid dimensions were used for dockings at the lipid facing surface residues (between the S6/S6 helices) (**Figure S1**). The coordinates used for the generation of the receptor grids for docking to the lipid facing surface was defined as a central point between the S5 and S6 helices (at F557 and M651T) for each four subunits. The GLIDE output provides a GScore (kcal/mol) which accounts for energy contributions from hydrophobic interactions, π-π stacking interactions between aromatic rings, root mean square deviation (RMSD), desolvation, protein-ligand interaction, and hydrogen bond formations. Only poses with energy score of ≤ −3 kcal/mol were used in further analysis. The absence of the membrane phase in docking studies represents a natural challenge to the direct interpretations of the binding scores and therefore we only use these scores to assess a relative likelihood (relative to the most favorable binding score) of different binding poses. The coordinates for the hERG1-dofetilide and hERG1-ivabradine complexes from docking studies are provided in the **Supplementary Information** section (receptor_drugs.zip). The full sets of docked conformations are available upon request.

### Molecular Biology Protocols

The site-directed mutagenesis methods utilized in this work have been previously reported (Lees-Miller et al., 2015; Wang et al., 2016). The hERG1 constructs were transfected into mammalian HEK cells. Conventional overlap PCR with primers were synthesized by Sigma Genosys (Oakville, Ontario, Canada) and sequenced by using Eurofins MWG Operon (Huntsville, AL) to create the single- and double-point mutant constructs of hERG1. Following this, *XbaI* restriction endonuclease was used to linearize and the cRNA was transcribed *in vitro* using the mMessage mMachine T7 Ultra cRNA transcription kit (Ambion, Austin, TX).

### General Setup for Electrophysiological Recordings

All experiments were performed under room temperature (295 K). The extracellular solution contained (mM) NaCl 140, KCl 5.4, CaCl_2_ 1, MgCl_2_ 1, HEPES 5, glucose 5.5; the pH of the solution was adjusted and kept at 7.4 with NaOH solution. Micropipettes were pulled from borosilicate glass capillary tubes using a programmable horizontal puller (Sutter Instruments, Novato, CA). The “intracellular” pipette solution contained the following: 10 mM KCl, 110 mM K-aspartate, 5 mM MgCl_2_, 5 mM Na_2_ATP, 10 mM EGTA [ethylene glycol-bis(-aminoethyl ether)-N,N,N,N tetraacetic acid], 5 mM HEPES, and 1mM CaCl_2_. The internal solution was then adjusted to pH 7.2 with KOH solution. Transfected HEK cells were patched to record the hERG1 currents. Standard patch-clamp methods were used to measure the whole cell currents of hERG1 mutants expressed in HEK 293 cells using the AXOPATCH 200B amplifier (Axon Instruments). Tail currents were recorded when the voltage was returned to −100 mV from +50 mV. Ivabradine was dissolved in Tyrode solution immediately before the experiments and the solutions were used for the next 2 h during the experiments. The stock solution of 100 µM ivabradine was prepared in the extracellular solution and fresh stock solutions of ivabradine were prepared weekly. To test the effect of intracellular application of dofetilide and ivabradine, drugs were applied to the intracellular space *via* the pipette. Dofetilide at 0.5 µM (~12 times greater than its IC50 value when applied extracellularly [0.04 µM]) was incorporated into the pipette solution. The intracellular block produced by dofetilide was compared to that of ivabradine at 100 µM, a value 17-fold greater than its IC50 value when applied extracellularly. The changes of hERG1 currents during the intracellular dialysis of the drug were monitored each minute right after forming the whole cell configuration.

### Statistical Analysis of Electrophysiological Experiments

Statsview (Abacus Concepts, Berkeley, CA), QTI plot (Vasilef, 2013), and Grace (http://plasma-gate.weizmann.ac.il/Grace/) were used to analyze the data. The null hypothesis of this study predicted no difference between the IC50 values comparing single to the double mutations. The null hypothesis was rejected at p < 0.05 as evaluated by a One-way Analysis of Variance with Tukey test. All variance measures (bars) for electrophysiological data are shown as Standard Deviation (SD). The study was exploratory; there was no *a priori* reason to consider whether there was an additive or subtractive interaction. The *n* values for each experiment are shown in **Figure 7**.

## Results

**Figure 3:**
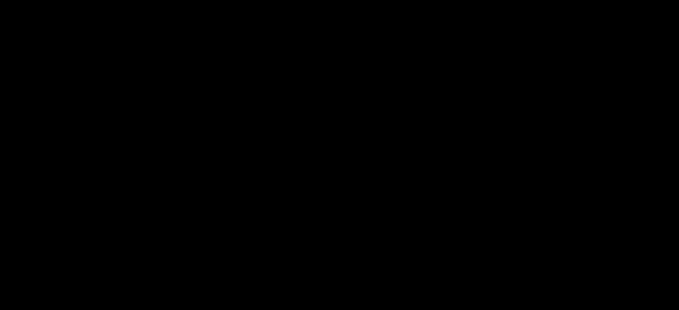
Ivabradine produces little block when applied intracellularly, *via* the pipette, even at concentrations 15-fold greater than its IC50 value when applied extracellularly. On the other hand, dofetilide when applied intracellularly managed to completely block the hERG1 current at 0.5 µM (a value of approximately 12-fold greater than its IC50 value, 0.04 µM, when applied extracellularly).

While the wild type hERG1 channel demonstrated dynamic F656 fluctuations in the cavity in micro-seconds long simulations, the M651T mutant “locked” the F656 residue in a specific configuration. We extracted several WT and M651T hERG1 structures from these simulations and explored the effects of the F656 conformation on drug binding. We also investigated the binding routes of dofetilide and ivabradine by combining mutagenesis and electrophysiology *in-vitro* data with docking to the MD-derived hERG1 structures (**Figures 1** and **2**). We demonstrate that dofetilide blocks the channel through the intracellular side of the membrane, indicating that the drug directly targets and blocks the central cavity, which is exposed to the intracellular fluid (**Figure 3**). In contrast, the preferential block of the WT-hERG1 by ivabradine occurs through the extracellular side of the membrane and little to no block of the channel is observed during the application of the drug from the intracellular side (**Figure 3**). Instead, ivabradine may be blocking the channel by partitioning from the extracellular leaflet towards the lipid-facing residues of the channel and then accessing and blocking the central cavity. We interrogated the *in-vitro* data presented here through docking simulations to unlock the residues involved in stabilizing the lipid-mediated pathway of ivabradine induced block of the channel.

### Bridging Docking With Electrophysiology

**Figure 4:**
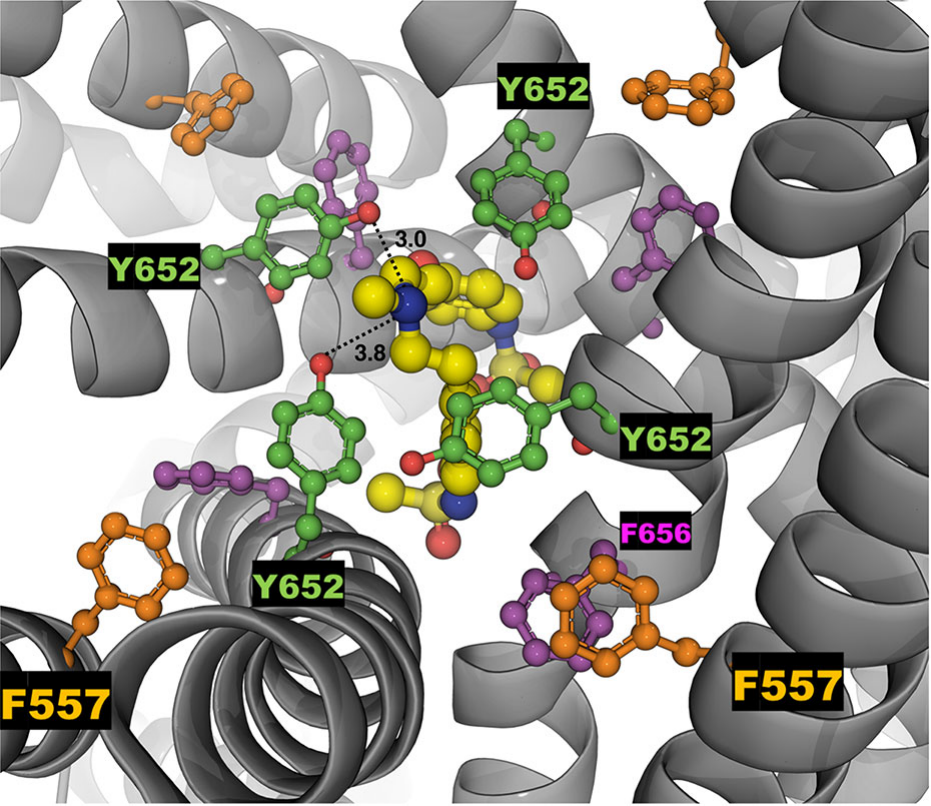
The top-ranked docked pose of neutral dofetilide (energy score of -5.1 kcal/mol) to the central cavity of the cryo-EM hERG1 (referred as state 1 in the text) is shown from the top perspective. The docked drug is shown in sticks and balls mode, while the receptor structure is shown as a gray cartoon. Key residues of the central cavity are labeled and represented as balls and sticks in different colors: Y652 (green), F557 (orange) and F656 (purple). The drug docked to the relaxed Cryo-EM state does not interact with F656 or F557. Distances between the -OH groups of Y652 and a central amine group of the dofetilide are measured in angstroms (Å) and shown as dashed lines.

Dofetilide and ivabradine were docked to the various pore-domain conformations of the WT and M651T hERG1 states identified in the analysis of MD simulations (**Figures 1** and **2**). We selected four states for WT-hERG1 and three states for the M651T mutants based on the side chain conformations adopted by F656 through time (**Figures 1** and **2**). Results of the energy scores (kcal/mol) from the docking of dofetilide and ivabradine to all states for all three binding locations are listed in **Table 1**. Our docking results consistently show that dofetilide binds to the MD derived WT-hERG1 states with lower energy scores in comparison to the starting cryo-EM equilibrated structure (state 1) (**Table 1**). The docking results with the cryo-EM equilibrated structure (state 1) yielded higher energy scores revealing unfavorable binding poses, most likely due to the upright F656 conformation in the structure (**Table 1**), in general agreement with the observations of Helliwell and colleagues (Helliwell et al., 2018). Dofetilide was consistently docked in a vertical position, which is too distant to interact with the aromatic rings of F656. Instead, the amine group of dofetilide was stabilized through interactions with the Y652 hydroxyl (-OH) groups (**Figure 4**). Drug docking outcomes to state 1 of the hERG1 channel do not parallel the results of the numerous experimental studies as F656 is crucial for dofetilide induced block of the hERG1 (Lees-Miller et al., 2000; Perry et al., 2004; Milnes et al., 2006; Saxena et al., 2016). Thus, poses of dofetilide found between the two S6 helices are highly unlikely. State 3 does not show binding of ivabradine in the cavity which does not correlate with experimental results reported in this paper and published previously (Melgari et al., 2015a; Lees-Miller et al., 2015), as the drug is sensitive to the mutations in the central cavity. State 4 demonstrates the nearly identical binding affinity for both drugs which also does not reflect our experimental data. Dofetilide is a “gold standard” hERG1 blocker, thus docking results are expected to yield a higher affinity to the cavity compared to ivabradine (Saxena et al., 2016). The docking results of the dofetilide and ivabradine to state 2 of hERG1 parallel and complement mutagenesis experiments in contrast to the cryo-EM equilibrated state 1 and the rest of the MD derived states suggesting that the F656 conformation of state 2 yields the highest affinity for the dofetilide induced block (**Table 1**). The following sections present our docking results to state 2 of hERG1 as it appears to be the most representative of the experimentally observed trends.

**Table 1:** Docking results of neutral ivabradine and dofetilde to four WT-hERG1 states to the central cavity and the lipid facing domain (between S6-S6 helices) are shown below. Drugs were found in the pore domain, lipid facing area or in-between S6-S6 helices.

		Pore domain	Lipid facing	In-between S6/S6
**State 1**	**IVA**	**−7.4**	**−5.9**	**−5.5**
	**DOF**	**−5.1**	**−5.7**	**—**

***State 2***	***IVA***	−5.8*	***−5.3***	***−4.6***
	***DOF***	***−7.0***	***−7.9***	**—**

**State 3**	**IVA**	**—**	**−4.6**	**—**
	**DOF**	**−7.1**	**−5.5**	**—**

**State 4**	**IVA**	**−8.1**	**−6.9**	**—**
	**DOF**	**−8.4**	**−5.0**	**—**

All numerical values are in (kcal/mol), Dashes represent poses undetected during the docking. State 2 is italicized as it is the most representative of the highest affinity open state hERG1.

*The best-scored ivabradine pose for state 2 corresponds to the position in the central cavity but slightly lower than a classical Y652–F656 pocket.

### The Central Cavity of the WT-hERG1 Is Flexible and Dynamic

**Figure 5:**
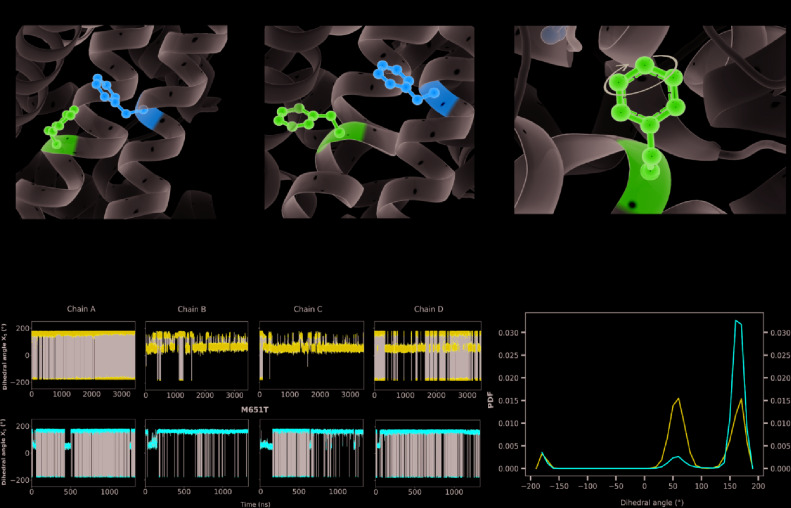
F656 flexibility is impaired in the M651T-hERG1. Two distinct conformational states of F656 (shown in purple sticks) interacting with F557 (orange sticks) are shown in **(A)**. The F656 is shown from the side perspective in a T-shaped interaction arrangement with the F557 at the time (Δt) ~0 µs corresponding to F656 side-chain torsional angle of χ_1_ ~170°. In the course of MD simulation F656 adopts “bent” into the central cavity conformation breaking T-shaped interaction with F557 illustrated with a state collected at the time (Δt) ~2.2µs. F656 side-chain torsional angle corresponding to this state is a χ_1_~50°. The time-traces of F656 χ_1_ for individual monomers in M651T and WT-hERG1 channels are shown in **(B)**. The total rotation of the F656 around its axis corresponding to changes in between χ_1_ ~−175° to χ_1_ ~+180° is illustrated in **(C)**. The Probability Distribution Function (PDF) for F656 dihedral angles show equal distribution of the "bent" (c1 ~170°) and “upright” ( c1 ~50°.) F656 configurations for the WT hERG1, whereas for the mutant, the bent configuration is almost diminished **(D)**.

Next, we assessed individual behaviors of F656, Y652, F557, and M651 for each chain of the protein from long MD simulations to analyze dynamics of individual residues involved in the formation of the hydrophobic cassettes in WT-hERG1 and M651T-hERG1. We measured side-chain χ_1_ dihedral angles (between C-Cα-Cβ-Cγ atoms) of the F656 and distances to Y652, F557, and M651 in all four chains. We observed that F656 is the most dynamic residue in the cavity contributing to the overall volume of the central cavity. The χ_1_ dihedral angles of the WT F656 shows that the F656 shifts from the upright position (c1 of ~ 175° or -185°) and bends towards Y652 in the central cavity, (c1 ~50°) for chains B, C, and D (**Figure 5**). The side chain of F656 of chain A of the WT-hERG1 exhibited significantly less mobility: it only rotated around its axis resulting in dihedral angles fluctuating between ~175° or −185° (**Figure 5**). These findings demonstrate the dynamic nature of the F656 conformational arrangement and how the F656 is coordinated by the surrounding residues.

### The M651T-hERG1 Mutant Rigidifies Central Cavity Due to Stabilization of F557–F656 Aromatic Stacking

**Figure 6:**
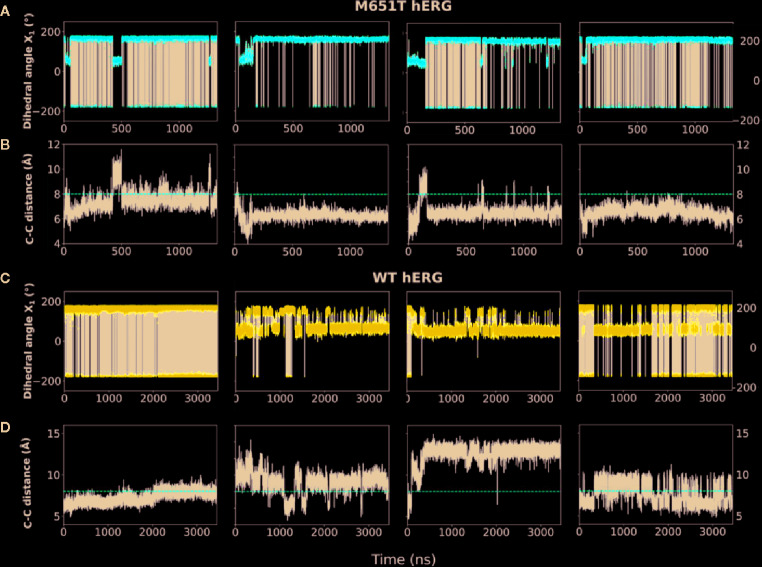
The F656-F557 stacking interaction between the aromatic rings is dominant in the M651T-hERG1 but not in the WT-hERG1. **(A–D)** Comparisons of time traces for F656 χ_1_ and F556–F656 C-C distances for the M651T-hERG1 (**A**, **B**, respectively) and the WT-hERG1 are shown (**C**, **D**, respectively). The time traces are averaged over all four monomers. The C-C distances corresponds to the distance between the two aromatic ring centers in F656 and F557. Dashed red line is at ~8 Å for ring-to-ring C-C distance corresponds to the state with F656 bent towards the water-filled intra-cellular cavity and T-shaped interaction between F656 and F557 is destabilized.

We investigated the effect of M651 mutation on the cavity dynamics by checking the surrounding residues. The frequency of the flipping of F656 is decreased in the M651T-hERG1 structure (**Figure 5D**). F656 is stabilized in an upright conformation through T-state π-π interactions with the aromatic rings of the F557 (**Figures 5A** and **6**). Distances of 4.5 Å between aromatic ring centers correspond to the most stable stacking interaction between the aromatic rings. The distance between the centers of the aromatic rings of the WT-hERG1 F656 and F557 remained at ~6–7 Å after a few nanoseconds of the equilibration. For the M651T-hERG1, a distance greater than 8 Å between F656 and F557 correlated with the bending of the F656 towards the inside of the central cavity and to significantly weaken interactions between the aromatic rings. The average distance between F557 and F656 was consistently shorter compared to the WT-hERG1 ~6.5 Å (**Figure 6B**). The interaction between the two residues (M651–F557) showed no correlation with F656 fluctuation dynamics as the distances for the residues in chains A, C, and D remained on average ~5 Å, and in chain B fluctuated between 5 Å and 10 Å (**Figure S2B**).

### Dofetilide Blocks Intracellularly by Binding to the Cavity of the WT-hERG1 and the M651T-hERG1

**Figure 7:**
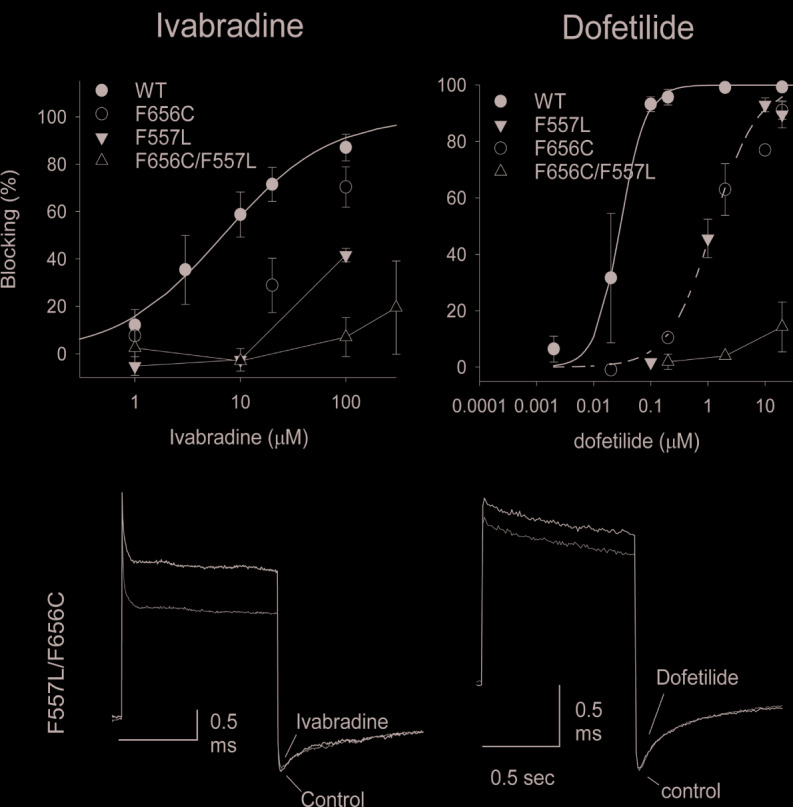
Concentration-response relationships for block produced by ivabradine and dofetilide for WT, F656C, F557L and for the double mutation F656C/F557L. The smooth curves were the successful fitting to Hill's equation. IC50 for ivabradine were 6.8 and 45 mM on WT and F656C, respectively **(A)**. IC50 for dofetilide were 0.029, 1.47, and 1.22 µM on WT, F656C, and F557L, respectively **(B)**. The n values for the experimental numbers shown in **(A)** were: n = 5, 5, 5, 5, 5 on WT in concentrations of 1, 3, 10, 20, 100 µM; n = 5, 5, 5 on F656C in 1, 20, 100 µM; n = 3, 4, 5 on F557L in 1, 10, 100 µM; n = 4, 2, 5, 2 on F656C/F557L in 1, 10, 100, 300 µM. The n values for the experimental numbers shown in **(B)** were n = 5, 5, 2, 6, 6, 4 on WT in 0.002, 0.02, 0.1, 0.2, 2, 20 µM; n = 1, 2, 6, 1, 3 on F656C in 0.002, 0.2, 2, 10, 20 µM; n = 3, 3, 3, 5 on F557L in 0.1, 1, 10, 20 µM; n = 2, 2, 4 on F656C/F557L in 0.2, 2, 20 µM, respectively. **(C, D)** Raw current traces. The superimposed current traces of F557L/F656C in control and 100 µM Ivabradine **(C)** or 2 mM dofetilide **(D)**. Note that since there was no block by these drugs that the control and drug traces overlap.

We next examined the impact of the single F557L, F656C and the double mutation (F557L/F656C) on the concentration-response relationships to ivabradine and dofetilide (**Figure 7**). We noted that the double mutation fully eliminated block by both dofetilide and ivabradine. In order to further establish the extent of loss of block, we applied suprapharmacologic concentrations of two drugs. We have previously reported both a high (100 nM) and a low affinity (1 µM) for binding of dofetilide (Duff et al., 1995). For the suprapharmacologic concentration of dofetilide, we applied a concentration of 20 µM (20-fold greater than the low affinity IC50 value) to be certain that near complete block would be seen for the wild type hERG1 channel. We also applied suprapharmacologic concentrations of ivabradine (100 µM). This concentration of ivabradine is 15-fold greater than its IC50 value (6.7 µM). Even at these very high concentrations, drug-induced block was eliminated in the double F557L/F656C mutation (**Figure 7**). Importantly, identical results were observed when dofetilide was applied at lower concentrations; either 0.5 µM or 2 µM (still suprapharmacologic). There is an additive effect of the double mutant F557L/F656C resulting in impaired blockade by dofetilide (~15% blocking compared to ~80% blocking for single mutants of F557 and F656). A similar trend is observed for ivabradine, resulting in ~15% blocking to the double mutant F557L/F656C (**Figure 7A**). In review, both dofetilide and ivabradine generated similar patterns of block in the F557L, F656C, and F557L/F656C hERG1 mutants. These results indicate that ivabradine and dofetilide interact similarly with F557 and F656 in the central cavity of the channel.

## Discussion

### F656 Flexibility Is Impaired in the M651T-hERG1

MD simulations have revealed that F656 exhibits several well-defined rotameric states alternating between the upright position observed in the initially equilibrated cryo-EM, a bent state in which the residue points into the central cavity and an outward pose where F656 is closer to the surrounding lipids (**Figures 1** and **2**). Our analysis suggests that the F656 dynamics in the WT-hERG1 are complex and depend on the intricate interplay of the aromaticity of the pocket with its surrounding residues including F557, M651, and Y652. During the simulation, direct interaction of F557, M651, and Y652 with F656 are observed. F557 forms π-π stacking interactions with F656 rings, and the sulfur atom of the M651 also may interact with the aromatic rings of the F656. Y652, which is located above the F656, may contribute to weak π-π stacking interactions with the F656 side chain. Together, these residues compete for interactions with F656. Another factor that contributes to the F656 geometric position in the central cavity is the limiting volume of the cavity. As the F656 and Y652 rings rotate and bend into the central cavity, steric hindrance may also be another contributing factor resulting in specific F656 configurations observed in states 2, 3, and 4. Overall, the interplay between these residues on the lipid facing domain (M651) and the S6 helices (F557, Y652) contribute to the fluctuating dynamic properties of the F656. The M651T-hERG1 results in more stable π-π interactions between F656 and F557 which maybe contributing to the loss of F656 flexibility (**Figure S3**). The cryo-EM configuration of the open-state hERG1 has been identified as a “low-affinity open state.” Some publications have reported attempts to manually shift the F656 from the upright position into the cavity-oriented state in order to obtain docking results that align with the experimental data. Even so, the cryo-EM structure only partly recapitulated the modelling data (Helliwell et al., 2018; Cernuda et al., 2019). Structures derived from the MD simulation may provide a reasonable docking model which does not require artificial manipulation of the structure and may illuminate possible drug binding poses as the structure of the channel naturally adopts a higher affinity open state as the flexible nature of F656 causes the residue to point towards the central cavity thereby allowing for stronger interactions with the blockers as shown in our work.

### Binding Pocket Aromaticity Is the Key Determinant in Dofetilide Block of the hERG1

**Figure 8:**
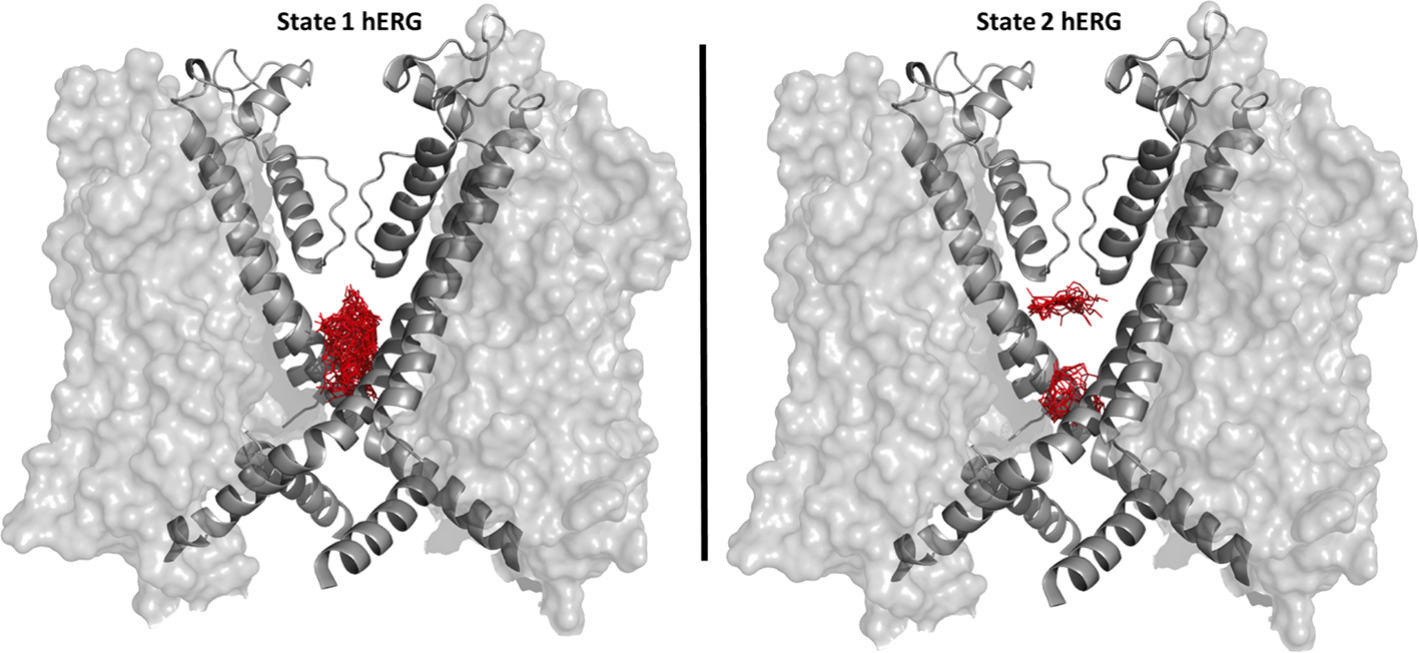
Distributions of docked poses for dofetilide is shown as sticks in red for the state 1 hERG1 (cryo-EM equilibrated) on the left side and state 2 MD derived hERG1 on the right side. For clarity purposes only the pore domain S6 helices of two subunits are shown as cartoons, the voltage sensing domain is represented as a transparent surface.

**Figure 9:**
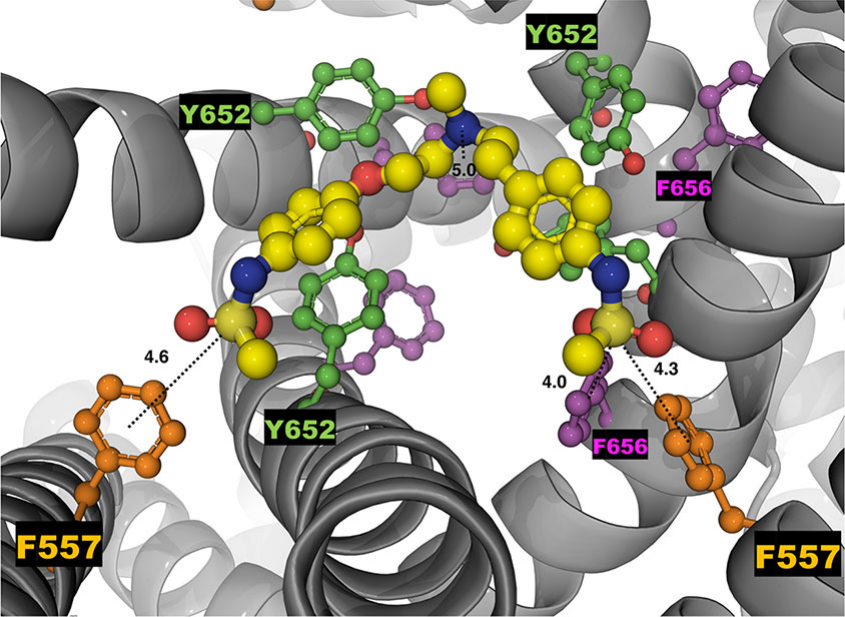
The top-ranked docked pose of neutral dofetilide (shown in yellow as sticks and balls) to the hERG1 state 2. The central cavity is shown from the top perspective. The top-ranked binding pose yielded an energy score of −7.0 kcal/mol. Sulfonamide groups of dofetilide extend towards the F557 from two subunits, measured distances in angstroms **(Å)** as dashed lines of 4.6 and 4.3 Å. The distance between the sulfur atom and the F656 on the right side is ~4.0 Å and the F656 with the central amine ~5 Å. Relevant residues are also shown as balls and sticks and labeled. Y652 is shown in green, F557 is in orange and F656 is in purple.

Dofetilide is sensitive to the mutation in the central cavity (F656, Y652), and to the F557 mutation in the S5. Our experimental results show that the double-point mutation F656C/F557L results in an almost complete loss of dofetilide block compared to the single-point mutations of F656 or F557, indicating an additive effect (**Figure 7**). The docking of dofetilide to the pore domain of the WT channel revealed the presence of two main binding sites: 1) hydrophobic pocket below the selectivity filter, near F557, F656, and Y652 and 2) a binding site below the F656 aromatic rings in a wider space (**Figure 8**). MD-derived states are better predictors of experimental measurements than the starting cryo-EM structure (**Figure 7**, **Table 1**). In the central cavity, dofetilide adopts a configuration in which the drug bends at the tertiary amine and the sulfonamide functional groups of the drug extend horizontally into the hydrophobic pockets, below the selectivity filter and above Y652 directly interacting with F557 (**Figure 9**). The flexibility of dofetilide at the tertiary amine is essential for the snug fit of the drug into this hydrophobic pouch. Interestingly, dofetilide analogues with higher rigidity around the aliphatic amino group of the drug resulted in reduced hERG1 block. The drug may potentially be losing the ability to fit into this binding site (Carvalho et al., 2013). Surrounding aromatic moieties in the cavity, specifically F656, mediate the direct interaction of dofetilide with F557. Sulfonamide groups of the drug interact with two of the F557 rings (~4 Å away), whereas the F656 aromatic rings (~5 Å away) stabilize the drug in the pocket (**Figure 9**). There are two F557 and two F656 residues that are involved in dofetilide stabilization in this pocket. The interplay between these residues contributes to the favorability of this configuration of the drug in this hydrophobic pouch. Ergo, aromaticity of this pocket was determined essential for drug stabilization in this binding spot. The multiple and stable rotameric states of F656 allow dofetilide to interact with the F557 and F656 simultaneously in support with the experimental data on dofetilide block through the intracellular milieu. Experimentally, the drug targets and finds the central cavity very quickly and favorably interacts with F656 and F557 (**Figures 3** and **9**).

### Ivabradine Block of the WT-hERG1 Occurs Through the Extracellular Milieu

**Figure 10:**
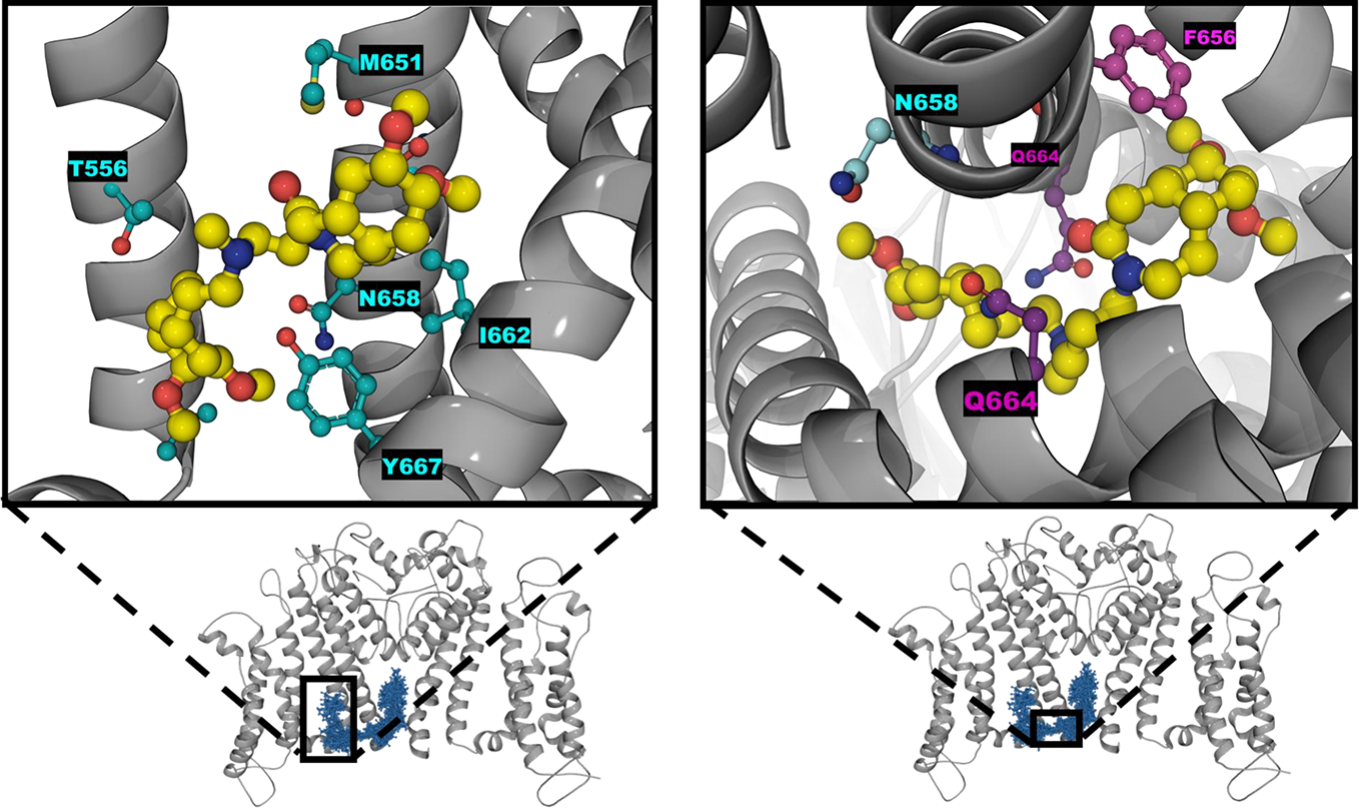
Zoomed-in configuration of ivabradine represented as sticks and balls (yellow) in the black boxes found on the lipophilic side shown on the left and inbetween the two S6 helices shown on the right. The overall protein is shown as cartoon alpha helices in gray with two subunits removed for clarity purposes. Distributions of the ivabradine poses are shown in blue as lines and sticks.

In this work, we show the ivabradine block of the hERG1 occurs from the extracellular milieu and little block of the channel is observed during the intracellular application of the drug (**Figure 3**). Ivabradine does not target the central cavity of the hERG1 directly from the intracellular side as efficiently as dofetilide, as shown by our experimental results (**Figure 3**). Instead, our docking results suggest ivabradine's block of the channel is mediated by the membrane facing residues of the channel (**Figure 10**). During the docking, the resulting poses were either in the pore, in the lipid facing surface, or in some cases, in-between the S6 helices (**Figure 10**). Residues that were in contact with the drugs within 5 Å of the lipid binding interface of the S5 and S6 include: M651, F557, L550, V549, Y667, I663, S654, N658, M554, G669, I647, F551. Ivabradine was also found to preferentially target a location between the segments of the S6-helices, we call the “in-between” pose (**Figure 10**). The plausible mechanism inferred from these data suggests a transition of the drug from the lipid-facing domain of the channel into central cavity as previously explored (**Figure S4**) (Perissinotti et al., 2019). The F656 stabilizes the aromatic ring of the ivabradine *via* π-π stacking interactions for the “in-between” poses. The drug was also interacting with the Y667 and Q664 from the adjacent subunits, as the drug was en-route to the central cavity.

### Lipophilic Binding Pathway of Ivabradine Is Disrupted by the M651T Mutant

The molecular simulations indicate that the M651T mutation affects the dynamics of F656, causing dramatically decreased instances of the “bent” configuration of χ_1_ ~50° (**Figure 5A**). Therefore, the cavity size of the M651T-hERG1 is larger than the WT-hERG1 as F656 does not point into the central cavity. The larger cavitary space of the mutant results in more energetically favorable binding of ivabradine in the cavity of the channel, as there are reduced instances of steric hindrance (**Table 2**). Given that the drug can favorably fit in the cavity, the binding route must be blocked in the M651T mutant as our previous experiments show (Perissinotti et al., 2019). Our docking showed no poses for the interface binding between the S6 for the M651T mutant states, suggesting the M651T mutant disturbs the entrance into the central cavity from the lipid facing side. Ivabradine is no longer “stabilized” in between the two S6 helices (**Figures 5** and **10**) because, the “bent” configuration of the aromatic ring in the WT-hERG1 is lost in the M651T mutant, and F656 is locked in the predominantly stationary conformation.

**Table 2:** The docking results for neutral states of ivabradine and dofetilide to three different states of M651T-hERG1 mutant to the central pore domain and the lipid facing domains (between S6-S6 helices). Poses of drugs were found either in the pore domain or the lipid facing domain and no drugs were in-between S6/S6 helices.

		Pore domain	Lipid facing	In-between S6/S6
**State 1**	**IVA**	**−8.7**	**−5.8**	**—**
	**DOF**	**−8.4**	**−4.5**	**—**

**State 2**	**IVA**	**−7.6**	**−4.7**	**—**
	**DOF**	**−7.4**	**−5.6**	**—**

**State 3**	**IVA**	**−10.0**	**−5.4**	**—**
	**DOF**	**−8.8**	**−4.4**	**—**

All numerical values are in kcal/mol.

## Conclusions

Our experimental and computational results suggest that ivabradine and dofetilide appear to block the central cavity of the channel *via* different paths. Our MD simulations coupled with docking and patch-clamp pipette experiments strongly support the notion that the aromaticity of the cavity governs the binding of dofetilide, whereas ivabradine requires fine-tuned conformational arrangement of the central cavity and utilizes the cavity's flexible nature for accessing the lipophilic pathway. Ivabradine is a larger molecule which requires a fine-tuned configuration of the central cavity to yield a favorable binding compared to dofetilide. The molecular modeling lend support to an idea that conformational plasticity of F656 is one of the determining factors in the cavity adaptation to a specific blocker. The conformational dynamics of aromatic cassette may play an important role in regulation of drug access to the main intra-cavity site. The conformational plasticity of the F656 residue is proposed to regulate the lipophilic entry pathway, which is lost in the mutant, M651T. The loss of conformational adaptation at F656 site in the mutant results in a significant decrease of ivabradine blockade.

## Data Availability Statement

The datasets generated for this study are available on request to the corresponding authors.

## Author Contributions

Participated in research design: JG, MK, JL-M, SN, and HD. Conducted experiments: JG, MK, FZ. Performed data analysis: JG, MK, HK, SN, and HD. Wrote or contributed to writing the manuscript: JG, MK, HK, SN, and HD.

## Funding

The work in SN's group was partially supported by the National Institutes of Health (USA) (Grant R01HL128537-03). SN and HD were supported by the Canadian Institutes for Health Research (Project Program FRN-CIHR: 156236). HK is supported by the Eyes-High Post-Doctoral Fellowship from the University of Calgary. FZ would like to acknowledge support from Summer 2019 Markin Undergraduate Student Research Program (USRP) in Health & Wellness. MK was supported by Bettina Bahlsen Memorial graduate scholarship, Queen Elizabeth II graduate scholarship and Jake Duerksen Memorial scholarship. Anton 2 computer time was provided by the Pittsburgh Supercomputing Center (PSC) through Grant R01GM116961 from the National Institutes of Health. The Anton 2 machine at PSC was generously made available by D.E. Shaw Research.

## Conflict of Interest

The authors declare the research was conducted in the absence of any commercial or financial relationships that could be construed as a potential conflict of interest.
