# Supplementary material for: Allosteric Coupling Between Drug Binding and the Aromatic Cassette in the Pore Domain of the hERG1 Channel: Implications for a State-Dependent Blockade
Source: Front Pharmacol. 2020 Jun 30;11:914. doi: 10.3389/fphar.2020.00914 (PMC7338687; doi:10.3389/fphar.2020.00914)

Supporting Information

Allosteric Coupling between Drug Binding and Aromatic Cassette in Pore Domain of hERG1 channel: Implications for A State-Dependent Blockade

Meruyert Kudaibergenova[1,2], Jiqing Guo[2], Hanif M. Khan[1], Farhan Zahid[1], James-Lee Miller[2], Sergei Yu. Noskov[1], Henry J. Duff [2]

[1]Centre for Molecular Simulation, Department of Biological Sciences, University of Calgary, Calgary, AB, Canada
[2]Libin Cardiovascular Institute of Alberta, Cumming School of Medicine, University of Calgary, Calgary, AB, Canada

The PDB coordinates for the receptor states 1 and 2 for WT and M651T hERG1 channels are provided in the supplementary materials along with the molecular geometries for top-ranked poses of cationic and neutral forms of dofetilide and ivabradine labeled DOF and IVA in the file names, respectively.

Table S1: Binding scores for the top 3 poses obtained for ivabradine and dofetilide to the pore domain of the hERG1 state1 and state2 show charge of the molecule doesn't affect the obtained scores. All numerical values are in kcal/mol, both neutral and charged states of the drugs were used in the docking to the central cavity of the channel.

WT hERG1	Dofetilide		Ivabradine	
	Neutral	Charged	Neutral	Charged
State 1	-5.1	-4.6	-7.4	-6.7
	-5.0	-4.5	-6.5	-6.1
	-3.6	-3.4	-6.0	-5.9
**Average**	**-4.6**	**-4.2**	**-6.6**	**-6.2**
State 2	-7.0	-8.2	-5.8	-7.9
	-6.9	-8.1	-5.0	-3.2
	-6.9	-6.3	-5.0	-3.1
**Average**	**-6.9**	**-7.5**	**-5.3**	**4.8**

Figure S1. Two overlapping molecular grids (on the right and left sides) that were used in our study are shown as boxes on the hERG1 receptors. The channel is represented as a colorful cartoon viewed from top to bottom. The inner box for XP docking is shown as a green box and an outer box is in purple. Left side depicts docking of the cavity of the channel, the right side shows the lipophilic coordinates used in docking between S6-S6 segments in proximity to M651.

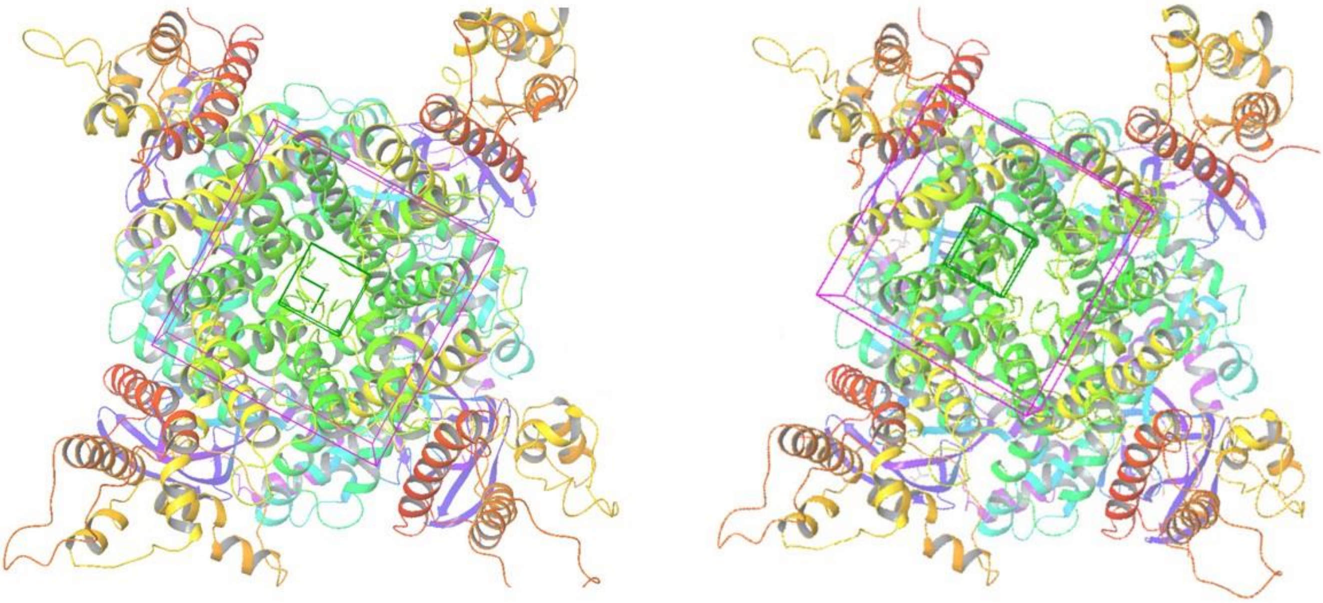

Figure S2. Time-traces for F557-M651 distances. Distances measured between the 557 and 651 positioned residues for the WT-hERG1 (Panel A) and for the M651T-hERG1 (Panel B). All four chains were used for calculations.

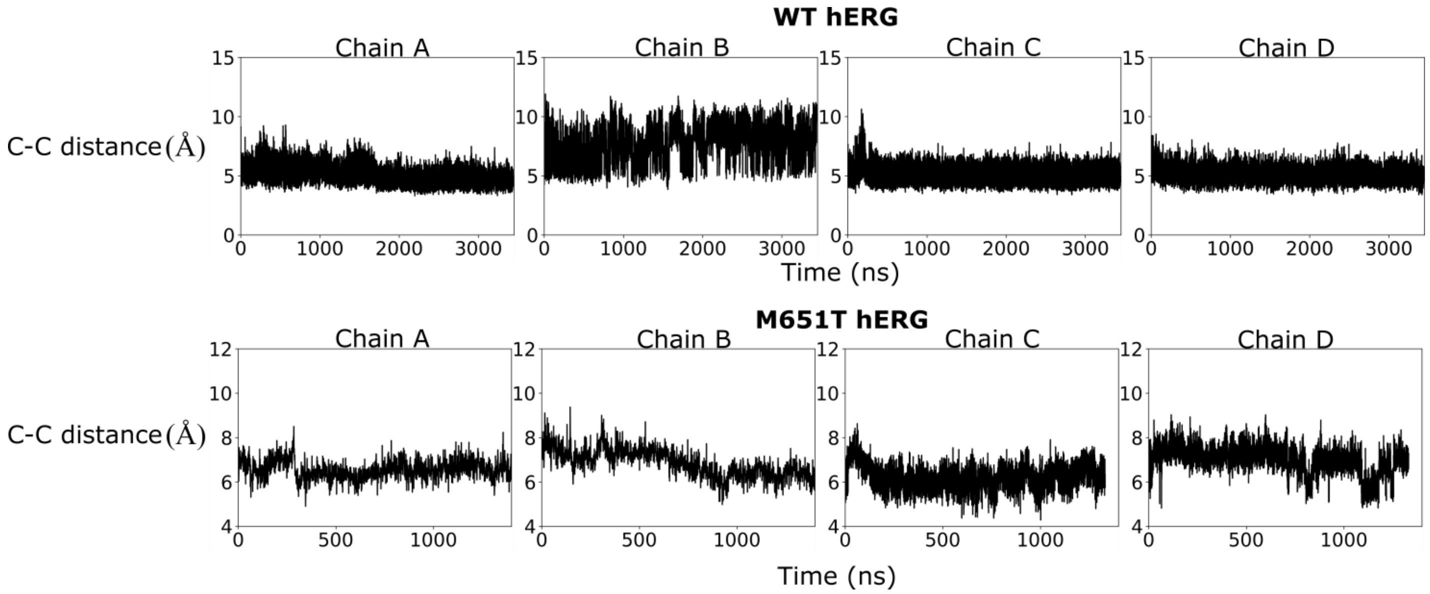

Figure S3. M651T enchances π-π interactions between F656 and F557. Representation of the relative location of the residues located at 651, the lipophilic site in the hERG1. WT-hERG1 (upper panel) and the M651T mutant (lower panel) pore domain and selectivity filter are shown as cartoons, the rest of the protein is represented as a surface for clarity purposes. Zoomed in perspectives are shown in purple boxes on the right side with F557, M651 and F656 positions. M651 is in teal for the WT-hERG1 and T651 for the M651T, F656 is in magenta and F557 is in orange.

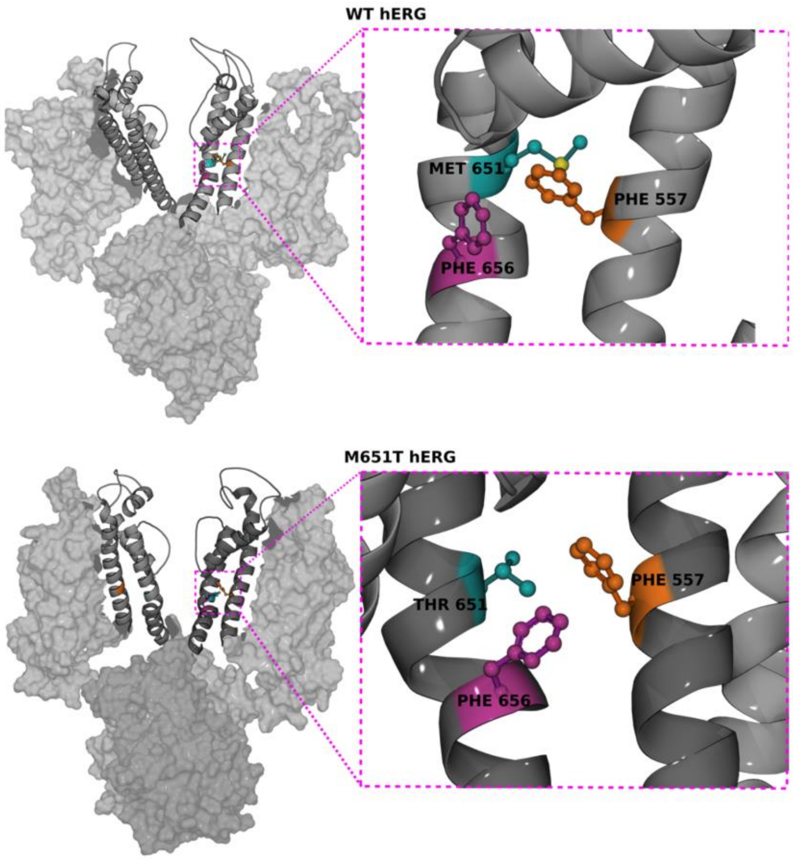

Figure S4. Proposed lipophilic binding pathway of ivabradine. Ivabradine induced block of hERG1 by accessing the central cavity through a lipid-mediated pathway from mapped from a → b → c. Perspective of the protein from the side projection, with opposing subunits removed for clarity purposes. Distribution of docked ivabradine poses shown in blue as sticks with 3 binding sites in dashed red lines. The lipophilic site (a), the in-between S6 helices (b), and the central cavity (c) on the WT state 2 of hERG1.

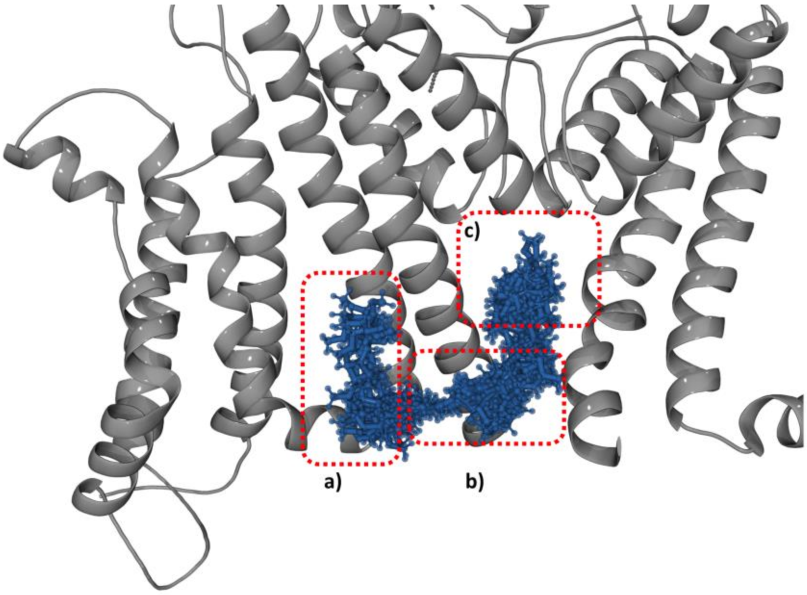

Supplement: Supplementary file 2 [file DataSheet_2.pdf]
